# Secondary Extramedullary Plasmacytoma of the Duodenum: An Unusual Endoscopic Presentation

**DOI:** 10.4021/gr547w

**Published:** 2013-07-14

**Authors:** Antonietta Gerarda Gravina, Alessandro Federico, Antonello Sica, Francesco Paolo D’Armiento, Maria Giovanna Ferrara, Umberto Falcone, Marcello Dallio, Domenico Cozzolino, Salvatore Guastafierro, Carmela Loguercio, Marco Romano

**Affiliations:** a Department of Clinical and Experimental Medicine, Gastroenterology Unit, Second University of Naples, Italy; b Onco-Hematology Units, Second University of Naples, Italy; c Department of Bio-morphological and Functional Sciences, University “Federico II” of Naples, Italy; d Internal Medicine, Second University of Naples, Italy; e These authors contributed equally to this work

**Keywords:** Extramedullary plasmacytoma, Duodenum, Diagnosis

## Abstract

We describe an unusual case (a 79-year-old woman) of secondary extramedullary plasmacytoma (EMP) involving the duodenum. While all the previously reported cases of duodenal involvement by EMP (namely 20 cases) were characterized by the presence of ulcerative masses or ischemic necrotic lesions, in our case EMP led to the unusual finding of several non-polypoid lesions with a depressed central area. The final diagnosis was multiple myeloma IgA lambda, stage II A, with secondary duodenal EMP. To our knowledge this is the first report showing duodenal involvement by EMP with the aspect of multiple non-polypoid lesions.

## Introduction

Extramedullary plasmacytoma (EMP) is a rare entity accounting for less than 4% of all plasma cell tumors and occurs mainly in the upper aero-digestive tract. In the GI tract most EMP occur in the stomach and only 20-30% arise in the small intestine [1]. Twenty cases involving duodenum, 24 involving jejunum and 17 involving ileum have been reported [2]. The involvement of duodenum is generally characterized by ulcerative masses or ischemic necrotic lesions or presence of infiltrating mass [3-5].

## Case Report

**Figure 1:**
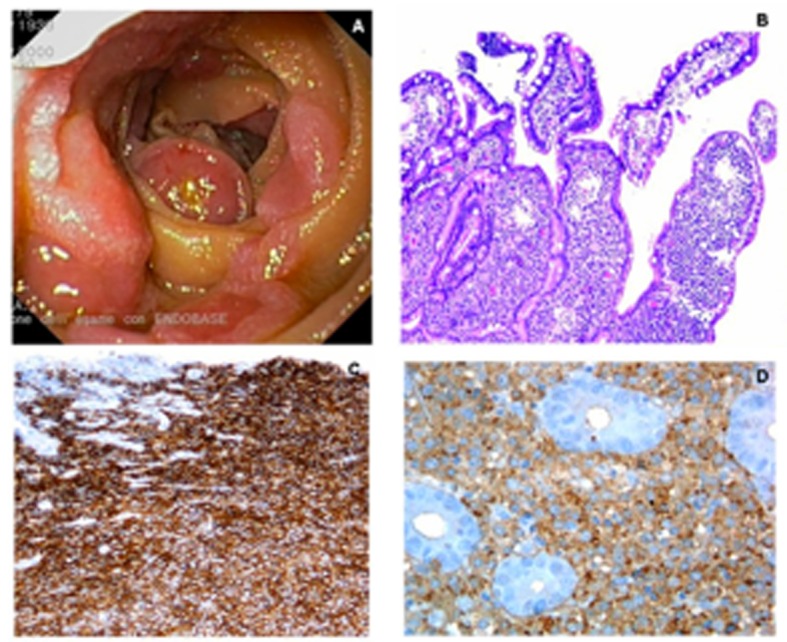
A. Multiple non-polypoid lesions in the second duodenum; B. *Infiltration of the villi by plasma cells with immature features (H&E, 400* ×*);* C. *Positive immunostaining for* the plasma cell marker *CD138 (400* ×*);* D. *Fine* membrane *granular* positivity for lambda immunoglobulin light chain (400 ×).

A 79-year-old woman was referred to our Gastroenterology Unit because of severe normocytic normochromic anemia (haemoglobin 6.8 g/dL) and positive fecal occult blood on three separate samples. Colonoscopy was negative. An upper gastrointestinal endoscopy was performed and showed antral erosive gastropathy without *H. pylori* infection at rapid urease test and histology. From the second to the fourth portion of the duodenum we found several grossly round non-polypoid lesions with a reddish central depressed area, II c + II a type, according to the Paris classification. The largest of these lesions had a diameter of about 1 cm (Fig. 1A). Multiple endoscopic biopsies specimens were taken and histology showed a plasma cell infiltrate characterized by immature plasma cells invading the submucosa (Fig. 1B). Immunohistochemistry was negative for cytokeratin, CD3 and CD5, T cell markers, CD20, a B cell marker, and neuroendocrine markers (S-100, vimentin, chromogranin, synaptophysin and CD56). Immunostaining was strongly positive for the plasma cell marker CD138 (Fig. 1C). Immunostaining for immunoglobulin lambda light chain was positive (Fig. 1D), while immunostaining for immunoglobulin kappa light chain was negative. The negative immunostaining for CD56 rules out a plasma cell neoplasm. The negativity for the B-cell marker CD20 ruled out the possibility of marginal zone B-cell lymphoma of mucosa associated lymphoid tissue. The morphology in conjunction with immunohistochemistry (CD138+/CD56-) was most consistent with an EMP consisting of poorly differentiated plasma cells. Serum electrophoresis and immunofixation showed an M spike of immunoglobulin A lambda. A bone marrow biopsy showed 36% of poorly differentiated plasma cells. Cytofluorimetry identified CD45dim+; CD38+, CD19-, CD56- plasma cells. Cytogenetics showed a normal female karyotype. Radiographic evaluation of the axial skeleton did not show osteolytic areas. The final diagnosis was multiple myeloma IgA lambda, stage II A, with secondary duodenal EMP.

## Discussion

To our knowledge this is the first report showing duodenal involvement by EMP with the aspect of multiple non-polypoid lesions. Moreover, in our case, it was the histology performed on duodenal biopsy specimens which led to the suspicion of multiple myeloma which was then confirmed at the subsequent histological evaluation of bone marrow. Our case report also points out the concept that the diagnosis of EMP should prompt a subsequent workup including quantitative and qualitative serum and urine protein analyses, a radiographic evaluation of the axial skeleton, and bone marrow biopsy. This is clinically relevant because therapy for primary or secondary EMP is quite different. Primary EMP often requires only radiotherapy or surgery, while secondary EMP requires systemic therapy.
